# Inconsistencies and Ambiguities in Liver-Disease-Related Contraindications—A Systematic Analysis of SmPCs/PI of Major Drug Markets

**DOI:** 10.3390/jcm11071933

**Published:** 2022-03-30

**Authors:** Laura Weisbach, Anna K. Schuster, Michael Hartmann, Martin F. Fromm, Renke Maas, Katrin Farker

**Affiliations:** 1 Hospital Pharmacy, University Center for Pharmacotherapy and Pharmacoeconomics, Jena University Hospital, Erlanger Allee 101, 07747 Jena, Germany; annakathrin.schuster@med.uni-jena.de; 2 Hospital Pharmacy, Jena University Hospital, Erlanger Allee 101, 07747 Jena, Germany; michael.hartmann@med.uni-jena.de; 3 Institute of Experimental and Clinical Pharmacology and Toxicology, Friedrich-Alexander-Universität Erlangen-Nürnberg, 91504 Erlangen, Germany; martin.fromm@fau.de (M.F.F.); renke.maas@fau.de (R.M.)

**Keywords:** liver disease, drug contraindications, liver-related contraindications, SmPC, PI, prescribing information, medication safety, drug market

## Abstract

Liver disease is a common condition worldwide that can cause alterations in drug disposition and susceptibility to drug toxicities, with increased risk of adverse drug reactions. European Summaries of Product Characteristics (SmPCs) and United States Prescribing Information (US PI) should therefore be comprehensible to prescribers regarding their liver-associated contraindications to ensure safe prescribing. This study aimed to evaluate the ambiguity of terminology used in communicating liver-associated absolute contraindications in SmPCs/PI of commonly prescribed drugs in four major drug markets (Germany, Switzerland, the United Kingdom, and the United States) by assigning wordings to different categories and analyzing their clinical comprehensibility. For US PI, 79% did not contain liver-related contraindications, compared to 2, 13, and 6% of German, Swiss, and British SmPCs, respectively. Study findings indicate that out of 228 examined SmPCs/PI containing liver-related contraindications, 77, 79, 76, and 52% contained unclear wording in the German, Swiss, British, and American drug market, respectively. Only 40% (German), 52% (Swiss), 39% (British), and 29% (American) of SmPCs/PI included terms with explicit wording. Including more precise statements in SmPCs/PI based on laboratory parameters (such as albumin) or scores (e.g., the Child–Pugh score) to objectify the severity of liver disease may improve the clarity of SmPCs/PI and the safety of drug prescription.

## 1. Introduction

Liver disease is a common condition worldwide that can cause alterations in drug disposition and susceptibility to drug toxicities, increasing the risk of adverse drug reactions. Liver function influences the pharmacokinetics and pharmacodynamics of a broad variety of drugs. Not only are the biotransformation and transport of drugs dependent on liver function, but so is the liver blood flow, while plasma protein binding and biliary excretion also affect drug disposition [1].

A review from 2013 estimated that 29 million persons in the European Union (EU) were suffering from a chronic liver condition and discussed the major causes leading to chronic liver disease. For example, hepatic cirrhosis (with alcohol as the most important risk factor) accounts for 170,000 deaths in Europe each year [2]. The United States (US) Center for Disease Control and Prevention (CDC) revealed a similar situation with cirrhosis-associated chronic liver disease, as it is the 12th leading cause of death and has a prevalence of nearly 15% [3]. In addition, drug-induced liver injuries (DILI) are a common adverse drug reaction. Paracetamol/acetaminophen overdosing is responsible for the largest proportion of cases of acute liver failure in developed countries [4,5]. Even without including such overdosing, more than 10% of all cases of acute liver failure are still identified as being associated with other drugs, biological agents, and dietary supplements [6].

Liver impairment not only has an impact on how different drugs function in the human body, but also how many drugs influence liver function and liver disease and therefore require relative or absolute contraindications in Summaries of Product Characteristics (SmPCs), often with monitoring or dose adjustment [7].

These considerations demonstrate the importance of accessible and clearly formulated prescribing information for healthcare professionals. Prescribing information in the EU is provided in the SmPCs and Package Leaflets (PL) standardized by the European Medicines Agency (EMA) [8]. American equivalents are the United States Prescribing Information (PI) and Patient Package Inserts backed by guidance of the US Food and Drug Administration (FDA). The content of these documents is provided by pharmaceutical companies and reviewed and published by the EMA/FDA during the authorization process. Nevertheless, each member state of the European Medicines Agency has the opportunity to authorize their own SmPC by a decentralized procedure, which can lead to different wordings for the same drug in different countries [9]. Moreover, standards are set by EU legislation and guidelines for PLs, including their comprehensibility, but not for SmPCs, which are generally required to be worded in “clear and concise language” according to the European Commission [10].

- **What is known:**
  Liver-related absolute contraindications are often described using terms with ambiguous wording and not clearly defined phrases, sometimes even used synonymously. A consensus for severity grading of liver-impairment-influencing pharmacokinetics and pharmacodynamics so far only exists for liver cirrhosis (Child–Pugh score).
- **What this study adds:**
  There is still need to improve unambiguousness in liver-related contraindications for prescribers. A possibility might be utilizing the already established consensus with the Child–Pugh score as a severity grading for mild, moderate, and severe liver impairment, and adding more examples to these severity grades using laboratory parameters (serum transaminases, albumin) or clearly defined diseases; also see Supplementary Table S4.
  - Severe hepatic impairment:
    ∘ liver cirrhosis, Child–Pugh score C
    ∘ acute hepatitis
    ∘ serum albumin < 25 g/L
    ∘ coma/precoma hepaticum
  - Moderate hepatic impairment
    ∘ liver cirrhosis, Child–Pugh score B
  - Mild hepatic impairment
    ∘ liver cirrhosis, Child–Pugh score A

Several studies examined inconsistencies and weaknesses of terminology in liver-related contraindications and prescribing recommendations [3,7,11]. For example, an often-used approach in the literature to assess the severity of hepatic impairment is to refer to the Child–Pugh score for hepatic cirrhosis, assigning different levels to *mild* (Child–Pugh score A), *moderate* (Child–Pugh score B), or *severe* (Child–Pugh score C) impairment of liver function [3]. However, a clearly specified definition of the severity of liver function impairment is usually lacking in the SmPCs, as reported by Weersink et al. [12] for 40% of 36 examined medicines. To describe the type of liver disease in SmPCs, four different but apparently interchangeable wordings were provided without further definition [12]. The observation that the term “liver impairment” may lack a clear definition in SmPCs is further supported by Bergk et al. [13]. Problems arise when liver function impairment is caused not by hepatic cirrhosis but other forms of liver disease, as there is no standardized grading for impaired liver function as there is with creatinine for assessing renal function [9,14]. This lack of a simple laboratory test causes uncertainty in prescribers about contraindications and dose adjustments for patients with liver disease [7].

Significant steps towards a more defined presentation of liver-related contraindications in product information reports were made in 2003 and 2005, when guidelines were published by the FDA and EMA to specify how research data for pharmacokinetics in liver disease patients should be presented [15].

Still, it remains uncertain which of the multitude of different terms used in SmPCs/PI to describe absolute contraindications regarding liver impairment is best suited to guide treatment in clinical practice. The aim of the present study is to assess the consistency and clinical comprehensibility of liver-related contraindications in SmPCs/PI of representative, commonly prescribed drugs in major drug markets (Germany (DE), Switzerland (CH), the United Kingdom (UK), and the US). Our intention was to point out common but unclear terms as a basis and an incentive for editing or clarification that leads to standardized definitions of contraindications in future SmPC/PI guidelines, so as to enhance safe medication prescribing and to facilitate the integration of this information in clinical decision support systems (CDSS).

## 2. Materials and Methods

**Figure 1:**
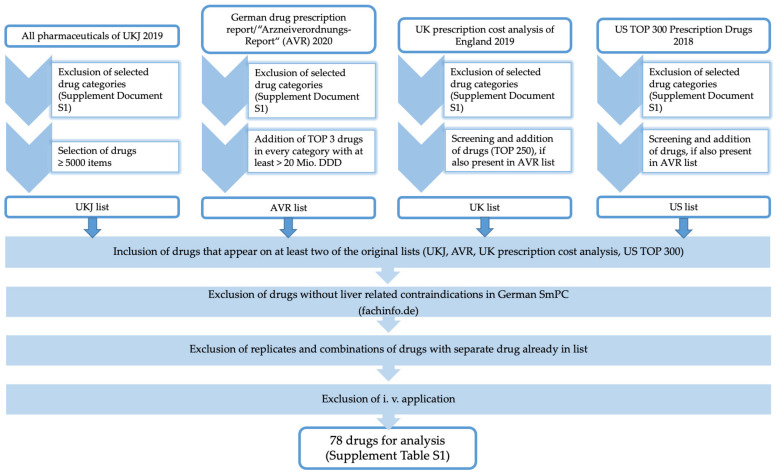
Selection of drugs for analysis.

Drugs assessed in the present analysis were selected as follows from four sources of the most-prescribed drugs in both hospital and ambulatory care (Figure 1). These included: [a] a list of all pharmaceuticals of the University Hospital Jena (UKJ), Germany, in 2019, [b] the 2020 German drug prescription report (Arzneiverordnungsreport, AVR) [16], which covers the German outpatient market for the statutory health insurances, [c] the prescription cost analysis of England, UK, in 2019 [17], and [d] the US top 300 prescription drugs of 2018 [18].

All drugs with no systemic effect, as well as vitamins, minerals, vaccines, insulins, antidotes, and veterinary and herbal medicines, among others, were excluded. The complete list of exclusion criteria is available in Supplementary Document S1, “Exclusion criteria of drug selection”, of this report. For the UKJ list, drugs that accounted for ≥ 5000 items (the quantity of pieces prescribed) were chosen. To include drugs from the AVR, which is the annual analysis of drug prescription in ambulant care in Germany (including the amount and the cost of the drugs prescribed) and is subdivided into indication groups/pharmacological effect mechanisms (categories), the top 3 drugs of every category (except excluded categories according to Supplementary Document S1 “Exclusion criteria of drug selection”) with at least > 20 Mio. Defined Daily Doses (DDD) were chosen to form the AVR list. Drugs from the UK prescription cost analysis (screening of the top 250) and top 300 US prescription drugs in 2018 were chosen for the UK and US list if they also appeared in the AVR list. In the next steps, drugs were chosen that appeared on at least two lists of the original sources. Using the directory of German SmPCs (“Fachinformationsverzeichnis Deutschland”) [19], those that did not contain an absolute liver-related contraindication in the German SmPC were excluded. Furthermore, replicates and combinations of drugs that were already on the list as a separate active ingredient and drugs for intravenous application were also excluded. The remaining 78 active ingredients form the final list; see Supplementary Table S1 “Selection of drugs for analysis”.

**Figure 2:**
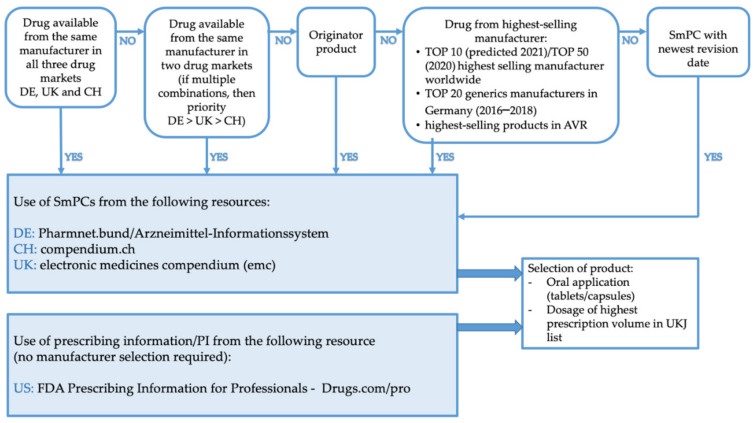
Selection of SmPCs/PI for analysis. References: TOP 10 (predicted 2021) [24], TOP 50 (2020) [25], TOP 20 generics manufacturers in Germany (2016–2018) [26], AVR [16].

For each of the 78 active ingredients, a product from a manufacturer including SmPCs from Germany, Switzerland, and the United Kingdom were chosen similarly to the method described by Pfistermeister et al., 2013 [20] (Figure 2). For this purpose, the databases pharmnet.bund/”Arzneimittel-Informationssystem”, which include products of all authorized drugs in Germany along with their SmPC [21], “Compendium.ch” for Swiss products and their SmPC [22], and the “Electronic Medicines Compendium (emc)” for products authorized in the UK and their SmPC [23] were used.

Products, and with them manufacturers, were chosen by the availability of their drug in the German, Swiss, and British drug markets, often representing manufacturers of originator products. When no product was available in all three of the markets, a product of a manufacturer was chosen that was available on two of the three drug markets with prioritization of availability for the German drug market first, followed by the British and then the Swiss drug markets. For products only available on one drug market, they were chosen in the following order: if possible, the originator product; second, the product of the highest selling manufacturer according to the top 10 (predicted in 2021) or top 50 (2020) highest selling manufacturers worldwide, and the top 20 highest selling generics manufacturers in Germany and the AVR [16,24,25,26]; and finally, the SmPC with the newest revision date. Selection was completed this way to avoid differences in SmPC wording by different manufacturers. US prescribing information was exclusively based on the ”FDA Prescribing Information for Professionals—Drugs.com” database [27], which does not require a selection of products but offers the newest prescribing information for active ingredients. Products and their dosages were selected for oral application (tablets, capsules) based on the product with the highest prescription volume of the UKJ list.

**Figure 3:**
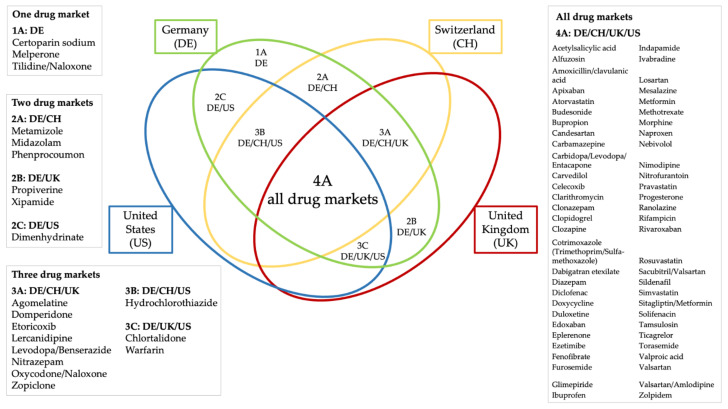
Overlap of active ingredients in the four drug markets.

These procedures resulted in a selection of 280 SmPCs/PI (78 products in the German market, 70 in the Swiss market, 70 British, and 62 US). Of note, not all of the 78 drugs available on the German market were available on all other markets (sometimes due to not being licensed elsewhere (e.g., metamizole) or not being licensed as a particular drug combination (e.g., oxycodone/naloxone)). Such variability accounts for the different numbers of SmPCs/PI/drugs in different markets (see also Figure 3, overlap of active ingredients in the four drug markets). One German SmPC also lacked an absolute liver contraindication even though active ingredients for the drug list were chosen by the presence of a liver-related absolute contraindication in the German SmPC on the homepage “fachinfo.de” (directory of German SmPCs). This circumstance can be explained by the process of selecting the active ingredients for the final drug list, as the selection was based on a product for oral administration without further selection criteria in a first screening step. After finalizing the drug list, their manufacturers were assigned as described above, so that different manufacturers may have been chosen from those of the products in the screening process. In this specific case (morphine), the screened SmPC contains the term “acute liver disease” as an absolute contraindication, whereas the chosen final product’s SmPC contains, among other comments, “severe hepatic impairment” in the “warnings” section. The complete list of analyzed drugs is available in Supplementary Table S1.

Liver-related terms and wordings in the SmPC/PI “Contraindications” sections and in the corresponding German “Gegenanzeigen”/”Kontraindikationen”, which contains absolute contraindications, were tabulated, assigned to different categories (Table 1 and Supplementary Table S2 “Category names and terms that were assigned to the respective categories. Complete list”), and analyzed for how many SmPCs/PI accounted for each category in the different drug markets. Each SmPC/PI was counted only once for each category, even if it contained multiple terms within one group. For example, an SmPC/PI containing the terms “severe hepatic impairment” and “moderate hepatic impairment, if…” was only counted once for the category “hepatic impairment”.

Within those categories, different terms were listed and evaluated for unambiguity and clarity. For this purpose, categories were assigned to three grades of explicitness regarding the terms they include, shown in Table 2. A wording was defined as explicit (Grade I) when an official definition for the disease, classification, or measurable conditions was described. Guidelines, disease classifications, and measurable laboratory parameters were considered valid as an official definition. Categories that contain understandable wording but leave open room for interpretation, and thus may lead to uncertainties, were assigned to the group of Grade II explicitness. Lastly, categories with unclear wording in their main expression were classified as Grade III. Diseases and associated conditions were evaluated separately. For each category, examples of relatively clear and explicit as well as unclear language were selected to outline the differences.

As the German and Swiss SmPCs are written in the German language, those phrases were translated in this paper, labeled with an asterisk (*), and the original term can be found in Supplementary Table S3 “Translation of German phrases used in the German or Swiss SmPCs translated into English”.

## 3. Results

### 3.1. Differences in the Number of SmPCs/PI with Liver-Disease-Related Contraindications in Different Products and Drug Markets

**Figure 4:**
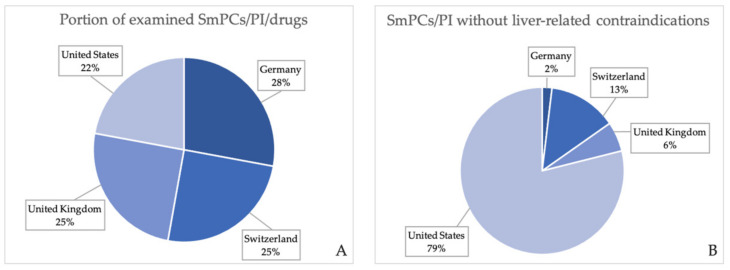
(**A**) Each drug market’s portion of the total amount of examined SmPCs/PI. (**B**) Each drug market’s portion of all examined SmPCs/PI without liver-related contraindications.

Figure 4A shows each market’s portion of the total amount SmPCs/PI that were included in the analysis. Considering the total of 280 SmPCs/PI of drugs which are approved in at least one of the four chosen drug markets, 28%, 25%, 25%, and 22% accounted for the German, Swiss, British, and US market, respectively, which corresponds to a balanced representation of SmPCs/PI from all markets. Out of the 52 SmPCs/PI without liver-related contraindications, only 2%, 13%, and 6% were authorized on the German, Swiss, and British market, respectively, representing an approximately even amount of SmPCs in the European drug markets compared to the remaining portion of 79% belonging to the US market (Figure 4B).

**Figure 5:**
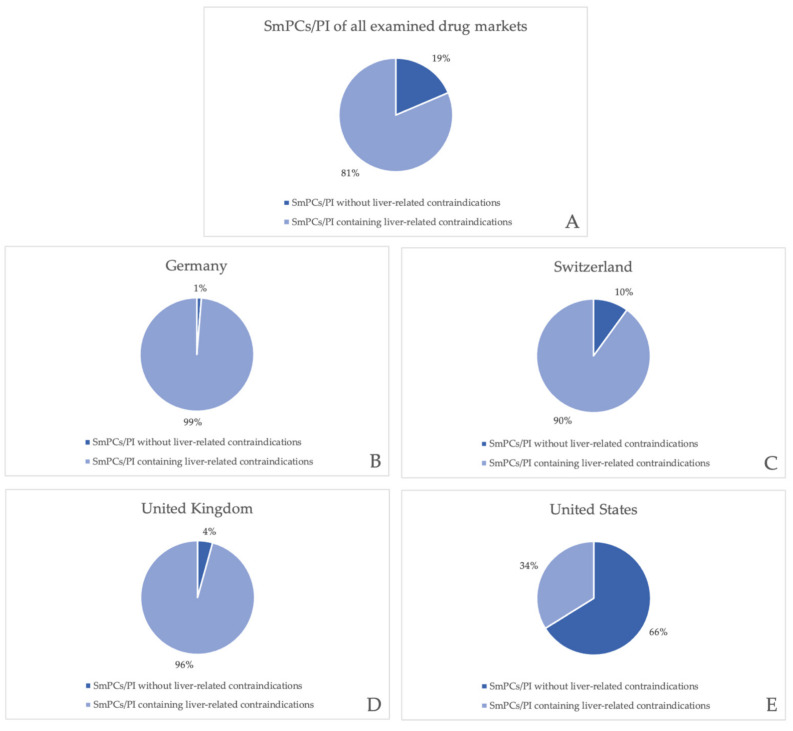
Portion of SmPCs/PI containing and without liver-related contraindications. (**A**) In the four chosen drug markets, (**B**) in Germany, (**C**) in Switzerland, (**D**) in the United Kingdom, and (**E**) in the United States.

As shown in Table 3, out of all 280 analyzed SmPCs/PI (78 products in the German, 70 products in the Swiss, 70 in the British, and 62 in the American market), a total of 52 SmPCs/PI did not contain an absolute contraindication regarding liver function or disease, which accounts for 19% of all SmPCs/PI (Figure 5A). Out of all examined European SmPCs, 99% of German, 90% of Swiss, and 96% of British SmPCs contained a liver-related contraindication, respectively (Figure 5B–D), showing only minor differences in the analyzed products. Compared to the German SmPCs, SmPCs that did not include a liver-related contraindication in the Swiss market (*n* = 7, 10%, Figure 5C) and the British market (*n* = 3, 4%, Figure 5D) contained liver function impairment/disease terms within another section, such as warnings, dose-adjustment advice, or pharmacokinetics. In contrast, the US drug market stands out in this comparison, in that out of all examined PI (*n* = 62), only 34% of the chosen drugs contained a liver-specific absolute contraindication as compared to the other drug markets (Figure 5E).

An additional finding is that, for some drugs, the presence of a liver-associated absolute contraindication differed between SmPCs/PI in different countries even though the same product and manufacturer were chosen for the analysis. For example, three drugs (valsartan/amlodipine, sacubitril/valsartan, and sitagliptin/metformin) contained liver-related absolute contraindications in German and British SmPCs, but not in the Swiss SmPC or the US PI. The SmPC of nimodipine included an absolute contraindication in the German but not the Swiss or British SmPCs, nor the American PI. Doxcycline had an absolute contraindication in the Swiss but not the British SmPC. In the other drug markets, only the SmPC of another manufacturer was available for the analysis of the last-mentioned drug (doxycycline).

### 3.2. Categories of Liver-Related Contraindication Wordings Account for Similar Relative Amounts of SmPCs/PI in All Drug Markets

Many different terms and even more associated conditions are used to describe liver-associated contraindications. To evaluate the wording, SmPCs/PI were assigned to different categories of liver disease/liver-related conditions. Therefore, wordings in SmPCs/PI were categorized according to the list in Table 1. Some categories account for several terms used synonymously. This applies to categories “albumin”, “ascites”, “Child–Pugh”, “hepatitis”, “hepatic failure”, “liver insufficiency”, “porphyria” and “transaminases”. Some categories contain fewer terms which cannot be used interchangeably, as they describe slightly different conditions within a wider generic term. These are “jaundice” (“obstructive jaundice”* as a subterm within the group of jaundice, but with different etiologies), “liver cirrhosis” (“biliary cirrhosis” as a subgroup within all cirrhotic diseases), and “precoma/coma”, which contains terms describing different stages of changes in consciousness related to liver impairment. The categories “hepatic impairment” and “liver disease” contain not only the highest number of assigned terms but also the most variety of conditions that can be defined by those wordings.

**Figure 6:**
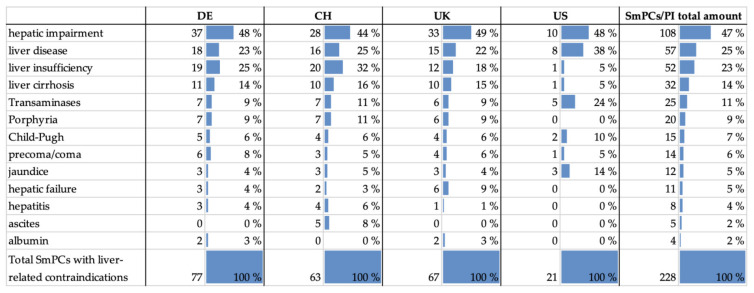
Number of SmPCs/PI with terms in assigned categories divided by drug markets.

Figure 6 shows the absolute number of SmPCs/PI in each country that contained one or more terms of the respective category, as well as their portion of the total number examined for the respective drug market. In addition to the absolute numbers, Figure 6 shows that, in total, the most SmPCs/PI were assigned to the categories “hepatic impairment” (*n* = 108, 47%), “liver disease” (*n* = 57, 25%), and “liver insufficiency” (*n* = 52, 23%), which contained expressions associated with them, whereas the fewest SmPCs/PI were found with expressions for the categories “albumin” (*n* = 4, 2%), “ascites” (*n* = 5, 2%), and “hepatitis” (*n* = 8, 4%). When divided by drug markets, the overall ranking was largely consistent in all countries for the amount of SmPCs/PI that were most often assigned with the respective categories of “hepatic impairment”, “liver disease”, “liver insufficiency”, and “liver cirrhosis”.

### 3.3. The Most Commonly Used Terms of Liver-Related Contraindications, “Hepatic Impairment”, “Liver Disease”, and “Liver Insufficiency”, Need More Clarification/Definition

The categories “hepatic impairment”, “liver disease”, and “liver insufficiency” are most frequently used in the the SmPCs/PI and have the broadest scope for interpretation. As the main term within the SmPC/PI is not clearly defined, supporting information is required for the correct interpretation, yet oftentimes the additional description leads to further uncertainty.

The group of terms associated with “hepatic impairment” is the most often used across all drug markets (47% of SmPCs/PI of all drug markets contained an expression of this group, Figure 6). The range of terms in this category includes expressions about liver/hepatic “impairment” and “dysfunction” in different wordings. Those were mostly accompanied by phrases regarding severity or examples of diseases that are included with the expression. These are sometimes useful for further concretizing the meaning, as they are clearly defined, e.g., “severe hepatic dysfunction (serum albumin < 25 g/L or Child–Pugh score ≥ 10)”, “severe hepatic impairment (liver cirrhosis and ascites)”*, and “with severe liver function impairment (for example liver cirrhosis)”*. On the other hand, added terms oftentimes do not contribute to a better understanding, but rather complicate clinical judgement, as the addition may not be clearly defined or the information may not be accessible. Examples are “liver function impairment with fatal outcome during therapy with valproic acid in siblings”*, “hepatic impairment or liver disease expected to have any impact on survival”, and “significantly impaired hepatic function”. The most often used terms, though, are “moderate” or “severe liver impairment“.

The second most frequently used category is “liver disease” (25% of all SmPCs/PI included a term of this group, Figure 6), which also contains different terms for “hepatic damage” and “liver tumors”*. Although “liver disease” is a clear expression to any prescriber, this group is comprised of a variety of diseases with very different clinical severities and different potentials for altering liver function with regard to drug effects. Therefore, this term is almost exclusively used in conjunction with further expressions. Unfortunately, associated wordings often do not improve understanding, but rather lead to more questions. Unclear or undefined additions to the term “liver disease” are, e.g., “active liver disease”, “liver disease resulting in hepatic impairment”, “clinical or biochemical evidence of significant liver disease”, and “acute or chronic liver disease (Rotor or Dubin-Johnson Syndrome”)*. Sometimes, additions to the main expression limit the field of possible conditions but still leave room for interpretation. Examples are: “active liver disease associated with nausea, anorexia or jaundice”, “hepatic disease associated with coagulopathy and clinically relevant bleeding risk including cirrhotic patients with Child Pugh B and C”, and “acute hepatic disease”.

The third most often used category in the three most prevalent category terms is “liver insufficiency” (23% of all examined SmPCs/PI contained wordings of this group, Figure 6). Like “hepatic impairment”, the expression “liver insufficiency” is a broad term applicable to a range of liver diseases of undefined clinical severity or types of functional liver impairment. A definition in the National Cancer Institute Thesaurus describes liver insufficiency as “the inability of the liver to perform its normal synthetic and metabolic functions” and as a synonym for liver failure [34]. Similar to expressions for hepatic impairment, liver insufficiency is oftentimes associated with further explanations or examples regarding severity. Further, as with the category “hepatic impairment”, “liver insufficiency” usually is paired with the grading “severe” or used without an additional description. The term liver insufficiency/severe liver insufficiency is not clearly defined. Other unclear expressions are combinations with the conditions “marked”*, “high-grade”*, “clinically manifest”* or “hepatic insufficiency (including biliary cirrhosis and unexplained persistent liver function abnormality)”. Enhancing the comprehensibility of this term are combinations, again the same as “hepatic impairment”, with the Child–Pugh score, e.g., “severe hepatic insufficiency (Child–Pugh-Class C)”, or laboratory parameters (“mild and moderate liver insufficiency (in children ALAT > 5 × ULN or bilirubin > 2 × ULN) with coagulopathy”*).

### 3.4. Categories Often Include Explicit as Well as Inexplicit Wording, Leaving Uncertainties

After assigning SmPCs/PI to different categories according to the defined terms, each category was analyzed for the explicitness of the category terms and the complete wording surrounding them. Some categories contain mostly explicit wording regarding the disease/condition of the category, as they are built on laboratory parameters, scores of defined classifications, or defined diseases (Grade I). Some categories involve understandable wording, but also contain uncertainties (Grade II); others have unclear wording (Grade III), see Table 2. Further, the comprehensibility of the complete wording in SmPCs/PI is highly dependent on the pairing of main conditions/diseases and associated conditions. Clearly defined categories such as “albumin”, “ascites”, “Child–Pugh” and partly “hepatitis”, “jaundice”, and “liver cirrhosis” are often not used to represent a contraindication, but rather to define or clarify less certain terms, such as liver disease/impairment/insufficiency.

Examples:

- “severe hepatic dysfunction (serum albumin < 25 g/L or Child–Pugh score ≥ 10)”
- “liver insufficiency (liver cirrhosis and ascites)”*
- “patients with severe hepatic impairment (for example acute hepatitis)”*

In addition, the clear definition of conditions, even those from clearly defined categories, may be weakened by associated conditions that contain unclear terms. For example, “Active liver disease or unexplained persistent elevations of serum transaminases” contains a possibly clearly understandable wording for transaminases, but fails to refer to a reference value (such as the often-used value “exceeding 3 × ULN”) or further limits comprehensibility with terms such as “unexplained and persistent” which are subjective and may not be assessable to the prescriber.

**Figure 7:**
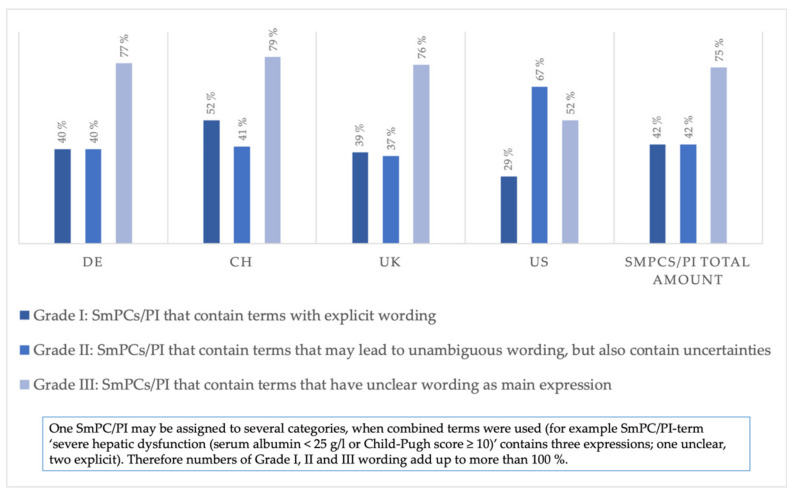
Portion of SmPCs/PI evaluated for explicitness of terms in all drug markets.

Figure 7 shows the distribution of SmPCs/PI within the three grades of clarity shown in Table 2. While 40, 52, 39, and 29% of German, Swiss, and British SmPCs and US PI, respectively, contain explicit wording/Grade I, 77, 79, 76, and 52% of German, Swiss, and British SmPCs and US PI, respectively, were classified as belonging to the category of unclear wording/Grade III. Moreover, 40, 41, 37, and 67% of German, Swiss, and British SmPCs and US PI, respectively, were assigned to the group of expressions that need more clarity/definition/Grade II. Note, that one SmPC/PI may be assigned to several categories when combined terms were used (see “examples” above).

### 3.5. Severity Grades That Are Not Clearly Defined Paired with Unambigous Information in SmPCs/PI Can Be Used as Guidelines for Severity Grading

When examining SmPCs/PI for their explicitness, oftentimes it is referred to as a severity grade (mostly “severe”) of an ambiguous liver-related condition, followed by better defined examples or clear laboratory parameters. Table 4 shows examples and laboratory values that are connected in SmPCs/PI. The best-defined grade of liver impairment is “severe”, which is defined in multiple SmPCs/PI by a Child–Pugh score C, serum albumin level, and an example of a disease (acute hepatitis), as well as defined symptoms (coma/precoma hepaticum). Moderate hepatic impairment can be defined via the Child–Pugh score B. Other examples better define ambiguous diseases but with no severity grading. For example, a frequent combination is “active liver disease” and “unexplained persistent elevations of serum transaminases exceeding 3 times the upper limit of normal”, which could be used to define the term “active liver disease”, but also leaves room for interpretation of the term “unexplained” for elevated transaminase levels.

## 4. Discussion

First, this comparison of the four selected drug markets found that the presence of liver-related contraindications did not differ much in the SmPCs of the same drug approved in the European drug markets, with a few exceptions. In contrast, fewer US PI of the same drugs contained liver-related absolute contraindications. The reason lies within the structure/composition of prescribing information in the US. Precautions in drug use regarding different diseases and conditions are often not stated as an absolute contraindication, but rather are explained in the warnings section with detailed advice on when to adjust dosage or avoid use of the drug, without completely prohibiting its use, leaving decision making to the prescriber. This finding validates a previous study by Nieminen et al. that examined the differences between US PI and SmPCs and also showed fewer contraindications in the US PI compared to European SmPCs due to their structure [35]. Another feature unique to American prescription information is the so called “boxed warning”/”black box warning”, which can also contain warnings without absolute contraindications but which emphasize the need to take specific conditions/diseases into account [36], but this was not analyzed in this study. Therefore, this analysis shows a balanced distribution of SmPCs with liver-related absolute contraindications for the European markets, but fewer US PI in comparison. Therefore, it should be taken into account that US PI expressions are underrepresented in the present study.

The second major finding is that the collection of terms that belong to one of the created categories for expressions regarding liver-related contraindications shows a similar distribution in all four drug markets. There may be several reasons for that. First, expressions for liver-related contraindications usually consisted of not just one but often several terms combined in a longer phrase. Therefore, categories with more generic terms contain more SmPCs/PI than categories with more exact wording and clear examples, as was also found in the distribution of SmPCs/PI within the category terms. This pattern may imply that SmPCs/PI follow a similar scheme of first using more general and less exact terms in all drug markets.

Furthermore, when these categories are divided into three groups to grade their explicitness, 75% of SmPCs/PI in all markets contain expressions belonging to the group comprised of the most ambiguous categories (Grade III). Often, no clear definition was provided for frequently used terms for liver impairment, insufficiency, or disease—terms used interchangeably, as previously noted by Weersink et al., 2019 [12]. An interesting question for further analysis would be whether the frequency of undesirable effects regarding the liver (for example in Section 4.8 of SmPCs) is connected to the way liver-related contraindications were presented. In the literature, so far there has been no research on a connection between the clarity regarding SmPCs (contraindications, warnings, and other sections) and liver-related side effects. Although extensive research has been conducted on DILIs, thereby inter alia resulting in the database LiverTox [37] with information on liver injury as an adverse drug reaction for a variety of prescription and nonprescription medications, the focus of those studies mainly lies in the evaluation of the drug causality and the identification of the drugs that most likely cause DILIs [38].

To improve the correspondence of clinical studies with the content of SmPCs/PI, guidelines for clinical studies provided by the EMA and FDA recommend to include patients with liver impairment and an assessment of their associated liver function. However, liver disease was only usually represented as liver cirrhosis in combination with a severity grading using Child–Pugh scores [39,40], which can be confirmed by the present study. Laboratory parameters such as serum concentrations of aminotransferases and bilirubin [41], similar to the criteria of DILI in the CIOMS consensus, are sometimes considered along with other methods to assess alterations in hepatic drug elimination using exogenous markers or probe drugs [39]. This practice explains how even though the term itself is not clearly defined as to whether liver disease or altered hepatic function classifies as “impairment”, the use of a classification such as the Child–Pugh score can provide greater clarity. For example, the following is an often-used consensus in the literature: “mild hepatic impairment” corresponds to liver cirrhosis, Child–Pugh A, “moderate hepatic impairment” corresponds to Child–Pugh B, and “severe hepatic impairment” to Child–Pugh C [42]. A first step in providing clear guidance for safer drug prescribing for patients with liver cirrhosis was published with Dutch recommendations by Stammschulte et al., categorizing often-used drugs into three groups to use for dose adjustment, utilizing the Child–Pugh score for liver cirrhosis [14]. Problems arise when any other liver disease is present but has not yet led to liver cirrhosis (e.g., acute hepatitis). For better medication safety, it would be advisable to create a consensus list of diseases for different categories and severity grades. Comprehensibility might be improved in SmPCs/PI when categories and concomitant conditions with explicit wording, especially using examples and laboratory parameters, could be grouped into the three severity grades of mild, moderate, and severe impairment of liver function. A first draft combining terms from Table 4 and categories from the Dutch recommendations can be found in Supplementary Table S4. Further, experts on liver disease might classify more diseases and/or terms that are already used in SmPCs/PI for those groups. Particular conditions that reduce the understanding of the main terms should be avoided or better defined.

Overall, this study demonstrates the extent of the still-existing incomprehensibility of liver-related absolute contraindications in SmPCs/PI in different drug markets and validates previous findings. These shortcomings underscore the need to reevaluate wordings in SmPCs/PI to help prescribers and manufacturers of CDSS improve the safety of drug prescription. Some efforts to enhance understanding are already in use including clearer severity grading, as with the Child–Pugh score, liver transaminase, or albumin levels. Even with such improvements, there remains a need for much more improvement, especially regarding phrases that tend to weaken rather than enhance the comprehensiveness of general terms. For future SmPC/PI guidelines, it is desirable to use terms based on defined criteria and international consensus to improve medication safety.

## Figures and Tables

**Table 1:** Category names and terms that were assigned to the respective categories. English terms only. Complete list (German and English terms) available in Supplementary Table S2.

Category	Terms
hepatic impairment	hepatic dysfunction, liver dysfunction, impairment of liver function, liver function abnormality, hepatic function disorder, decompensated hepatic function, impaired hepatic function, impairment of hepatic function
liver disease	hepatic disease, hepatic damage
liver insufficiency	hepatic insufficiency
liver cirrhosis	cirrhosis, biliary cirrhosis, hepatic cirrhosis
transaminases	serum transaminases
porphyria	no additional terms
Child–Pugh	Child–Pugh class/score
precoma/coma	hepatic coma, hepatic encephalopathy, precomatose states (associated with liver cirrhosis)
jaundice	no additional terms
hepatic failure	no additional terms
hepatitis	no additional terms
ascites	no additional terms
albumin	serum albumin

**Table 2:** Categories evaluated for explicitness of terms.

Grade I: Categories with explicit wording	“albumin”, which refers to laboratory parameters with a clearly defined laboratory value “ ascites”, defined symptom (liver-associated diagnostics and therapy, see Updated S2k-Guideline “Complications of liver cirrhosis” [28]) “Child–Pugh”, defined score for liver cirrhosis [29] “ hepatitis”, defined disease [30] “ jaundice”, defined symptom [30] “ liver cirrhosis”, defined disease [30] “ porphyria”, defined disease [30]
Grade II: Categories that may lead to unambiguous wording, but also contain uncertainties	“coma/precoma”, partly defined by West-Haven-Criteria, but precomatose states are not clearly defined [31] “liver disease”, broad spectrum of diseases, that can be defined but no reference for inclusion or exclusion of certain conditions, as this term may address functional (i.e., metabolic and pharmacokinetic) aspects as well as the organ vulnerability to toxic effects [30] “transaminases”, refers to laboratory parameters of aspartate transaminase (AST) and alanine transaminase (ALT), but sometimes no defined values noted
Grade III: Categories that have unclear wording as main expression	“hepatic impairment”, no definition “hepatic failure”, attempts of definition, but may contain different types of liver failure, that are not referred to [32,33] “liver insufficiency”, no clear definition [34]

**Table 3:** Total number of SmPCs/PI (*n* = 280) divided into those containing liver-associated contra-indications (*n* = 228) and those not containing liver-associated contraindications (*n* = 52) in the four chosen drug markets.

Country	Number of Examined SmPCs/PI/Drugs in Each Country	SmPCs/PI Containing Liver-Related Contraindications	SmPCs/PI without Liver-Related Contraindications
Germany	78	77 (99%)	1 (1%)
Switzerland	70	63 (90%)	7 (10%)
United Kingdom	70	67 (96%)	3 (4%)
United States	62	21 (34%) ^1^	41 (66%) ^1^
Total number of SmPCs/PI	280 ^2^	228 (81%)	52 (19%)

^1^ The difference in the amount of SmPCs/PI containing/not containing liver-related contraindications in the US compared to other drug markets is explained in the text. ^2^ The portion of the total number of examined SmPCs/PI for each country: Germany 28%, Switzerland 25%, United Kingdom 25%, United States 22% (see also Figure 4A).

**Table 4:** Combinations of ambiguous and more clearly defined terms.

Term	Disease/Laboratory Parameter	Original Term in SmPC/PI
Severe hepatic impairment	Liver cirrhosis, Child–Pugh score C	Severe hepatic dysfunction (serum albumin < 25 g/L or Child–Pugh score ≥ 10) *
Severe hepatic impairment	Acute hepatitis	Patients with severe hepatic impairment (for example acute hepatitis) *
Severe hepatic impairment	Serum Albumin < 25 g/L	Severe hepatic dysfunction (serum albumin < 25 g/L or Child–Pugh score ≥ 10) *
Severe hepatic impairment	Coma/Precoma hepaticum	Severe hepatic impairment with coma/precoma hepaticum *
Severe hepatic insufficiency	Liver cirrhosis, Child–Pugh score C	Patients with severe hepatic insufficiency (Child–Pugh Class C)
Moderate hepatic impairment	Liver cirrhosis, Child–Pugh score B	Patients with moderate or severe hepatic impairment (Childs-Pugh categories B and C)

* Translated term from German to English. The original term can be found in Supplementary Table S3 “Translation of German phrases used in the German or Swiss SmPCs translated into English”.

## Supplementary Materials

The following are available online at https://www.mdpi.com/article/10.3390/jcm11071933/s1, Document S1: Exclusion criteria of drug selection; Table S1: Selection of drugs for analysis; Table S2: Category names and terms that were assigned to the respective categories. Complete list; Table S3: Translation of German phrases used in the German or Swiss SmPCs translated into English; Table S4: Examples of assigning conditions to severity grades.

## References

[1] Verbeeck R.K. (2008). Pharmacokinetics and dosage adjustment in patients with hepatic dysfunction. Eur. J. Clin. Pharmacol..

[2] Blachier M., Leleu H., Peck-Radosavljevic M., Valla D.C., Roudot-Thoraval F. (2013). The burden of liver disease in Europe: A review of available epidemiological data. J. Hepatol..

[3] Chang Y., Burckart G.J., Lesko L.J., Dowling T.C. (2013). Evaluation of hepatic impairment dosing recommendations in FDA-approved product labels. J. Clin. Pharmacol..

[4] Stravitz R.T., Lee W.M. (2019). Acute liver failure. Lancet.

[5] Andrade R.J., Chalasani N., Björnsson E.S., Suzuki A., Kullak-Ublick G.A., Watkins P.B., Devarbhavi H., Merz M., Lucena M.I., Kaplowitz N. (2019). Drug-induced liver injury. Nat. Rev. Dis. Prim..

[6] Council for International Organizations of Medical Sciences (CIOMS) (2020). Drug-Induced Liver Injury (DILI): Current Status and Future Directions for Drug Development and the Post-Market Setting.

[7] Hayward K.L., Weersink R.A. (2020). Improving medication-related outcomes in chronic liver disease. Hepatol. Commun..

[8] A Guideline on Summary of Product Characteristics (SmPC). https://ec.europa.eu/health/sites/default/files/files/eudralex/vol-2/c/smpc_guideline_rev2_en.pdf.

[9] Bjornsson E.S., Jacobsen E.I., Einarsdottir R., Chalasani N. (2015). Discrepancies in liver disease labeling in the package inserts of commonly prescribed medications. Gastroenterology.

[10] Raynor D.K., De Veene P., Bryant D. (2014). The effectiveness of the summary of product characteristics (SmPC) and recommendations for improvement. Ther. Innov. Regul. Sci..

[11] Weersink R.A., Burger D.M., Hayward K.L., Taxis K., Drenth J.P.H., Borgsteede S.D. (2020). Safe use of medication in patients with cirrhosis: Pharmacokinetic and pharmacodynamic considerations. Expert Opin. Drug Metab. Toxicol..

[12] Weersink R.A., Timmermans L., Monster-Simons M.H., Mol P.G.M., Metselaar H.J., Borgsteede S.D., Taxis K. (2019). Evaluation of information in summaries of product characteristics (SmPCs) on the use of a medicine in patients with hepatic impairment. Front. Pharmacol..

[13] Bergk V., Haefeli W.E., Gasse C., Brenner H., Martin-Facklam M. (2005). Information deficits in the summary of product characteristics preclude an optimal management of drug interactions: A comparison with evidence from the literature. Eur. J. Clin. Pharmacol..

[14] Stammschulte T., Weersink R., Sauerbruch T., Poralla T., Farker K., Köberle U., Borgsteede S.D. (2020). Niederländische empfehlungen zur sicheren anwendung von arzneimitteln bei leberzirrhose. Arzneiverordn. Prax..

[15] Weersink R.A., Bouma M., Burger D.M., Drenth J.P.H., Harkes-Idzinga S.F., Hunfeld N.G.M., Metselaar H.J., Monster-Simons M.H., Taxis K., Borgsteede S.D. (2018). Evidence-based recommendations to improve the safe use of drugs in patients with liver cirrhosis. Drug Saf..

[16] Schwabe U., Ludwig W.D. (2020). Arzneiverordnungs-Report 2020.

[17] UK Prescription Cost Analysis England 2019. https://www.nhsbsa.nhs.uk/statistical-collections/prescription-cost-analysis-england/prescription-cost-analysis-england-2019.

[18] The Top 300 of 2019. https://clincalc.com/DrugStats/Top300Drugs.aspx.

[19] Directory of German SmPCs (Fachinformationsverzeichnis Deutschland). https://www.fachinfo.de/.

[20] Pfistermeister B., Schenk C., Kornhuber J., Bürkle T., Fromm M.F., Maas R. (2013). Different indications, warnings and precautions, and contraindications for the same drug-an international comparison of prescribing information for commonly used psychiatric drugs. Pharmacoepidemiol. Drug Saf..

[21] Arzneimittelinformationssystem Deutschland. https://portal.dimdi.de/amguifree/am/search.xhtml.

[22] Compendium.ch. https://compendium.ch/.

[23] Electronic Medicines Compendium (EMC). https://www.medicines.org.uk/emc/.

[24] Top10 Highest-Selling Manufacturers Worldwide 2021. https://de.statista.com/statistik/daten/studie/246872/umfrage/-umsatzstaerkste-pharmaunternehmen-weltweit/.

[25] TOP50 Highest-Selling Manufacturer Worldwide 2020. https://de.statista.com/statistik/daten/studie/439880/umfrage/top-50-pharmaunternehmen-umsatz-und-forschungsausgaben/.

[26] TOP Generics Manufacturers in Germany 2016–2018. https://de.statista.com/statistik/daten/studie/678895/umfrage/marktanteile-fuehrender-generikahersteller-in-deutschland-nach-umsatz/.

[27] FDA Professional Drug Information. https://www.drugs.com/pro/.

[28] Gerbes A.L., Labenz J., Appenrodt B., Dollinger M., Gundling F., Gülberg V., Holstege A., Lynen-Jansen P., Steib C.J., Trebicka J. (2019). Updated S2k-guideline ‘Complications of liver cirrhosis’. German society of gastroenterology (DGVS). Z. Gastroenterol..

[29] Child C.G., Turcotte J.G. (1964). Surgery and portal hypertension. Major Probl. Clin. Surg..

[30] Herold G. (2021). Innere Medizin 2021.

[31] Weissenborn K. (2019). Hepatic encephalopathy: Definition, clinical grading and diagnostic principles. Drugs.

[32] Wendon J., Cordoba J., Dhawan A., Larsen F.S., Manns M., Nevens F., Samuel D., Simpson K.J., Yaron I., Bernardi M. (2017). EASL clinical practical guidelines on the management of acute (fulminant) liver failure. J. Hepatol..

[33] Arroyo V., Moreau R., Jalan R. (2020). Acute-on-chronic liver failure. N. Engl. J. Med..

[34] National Cancer Institute Thesaurus. https://ncit.nci.nih.gov/ncitbrowser/pages/home.jsf;jsessionid=FCF0FA1B6461DFB54F3D0843E3C6B644.

[35] Nieminen O., Kurki P., Nordström K. (2005). Differences in product information of biopharmaceuticals in the EU and the USA: Implications for product development. Eur. J. Pharmaceutics Biopharm. Off. J. Arbeitsgem. Pharm. Verfahrenstech. e.V..

[36] FDA Guidance Document. https://www.fda.gov/regulatory-information/search-fda-guidance-documents/warnings-and-precautions-contraindications-and-boxed-warning-sections-labeling-human-prescription.

[37] LiverTox. https://www.ncbi.nlm.nih.gov/books/NBK547852/#IX-A.

[38] Douros A., Bronder E., Andersohn F., Klimpel A., Thomae M., Sarganas G., Kreutz R., Garbe E. (2015). Drug-induced liver injury: Results from the hospital-based Berlin case-control surveillance study. Br. J. Clin. Pharmacol..

[39] EMA Guideline on the Evaluation of the Pharmacokinetics of Medicinal Products in Patients with Impaired Hepatic Function. https://www.ema.europa.eu/en/documents/scientific-guideline/guideline-evaluation-pharmacokinetics-medicinal-products-patients-impaired-hepatic-function_en.pdf.

[40] Guidance for Industry (fda.gov). https://www.fda.gov/media/71311/download.

[41] Clinical Protocol Rivaroxaban Bayer HealthCare AG and Janssen Pharmaceuticals. https://clinicaltrials.gov/ProvidedDocs/78/NCT02555878/Prot_001.pdf.

[42] Talal A.H., Venuto C.S., Younis I. (2017). Assessment of hepatic impairment and implications for pharmacokinetics of substance use treatment. Clin. Pharmacol. Drug Dev..

