# Supplementary material for: Inconsistencies and Ambiguities in Liver-Disease-Related Contraindications—A Systematic Analysis of SmPCs/PI of Major Drug Markets
Source: J Clin Med. 2022 Mar 30;11(7):1933. doi: 10.3390/jcm11071933 (PMC9000103; doi:10.3390/jcm11071933)
Supplement: Supplementary file 1 [file jcm-11-01933-s001.zip › Table S3.pdf]

Table S3: Translation of German phrases used in the German or Swiss SmPCs translated into English

Terms that were translated from German into English in this paper, marked with asterisk (*).

Translation in text	Term in SmPC
obstructive jaundice	Verschlussikterus
severe hepatic impairment (liver cirrhosis and ascites)	Schwere Leberfunktionsstörungen (Leberzirrhose und Aszites)
with severe liver function impairment (for example liver cirrhosis)	mit schwerer Leberfunktionsstörung (z. B. Leberzirrhose
liver function impairment with fatal outcome during therapy with valproic acid in siblings	Leberfunktionsstörungen mit tödlichem Ausgang während einer Valproinsäuretherapie bei Geschwistern
liver tumors	Lebertumore
Acute or chronic liver disease (Rotor- or Dubin-Johnson-Syndrome)	akute oder chronische Leberleiden (Rotor- oder Dubin-Johnson-Syndrom)
marked	ausgeprägte
high-grade	hochgradige
clinically manifest	klinisch manifeste
mild and moderate liver insufficiency (in children ALAT > 5 x ULN or bilirubin > 2 x ULN) with coagulopathy	leichter und mässiggradiger Leberinsuffizienz (bei Kindern ALAT > 5x ULN oder Bilirubin > 2x ULN) in Kombination mit Koagulopathie
hepatitis	Hepatitis
liver insufficiency (liver cirrhosis and ascites)	Leberinsuffizienz (Leberzirrhose und Aszites)
patients with severe hepatic impairment (for example acute hepatitis)	Patienten mit schwerer Leberfunktionsstörung (z. B. akute Hepatitis)
severe hepatic impairment with coma/precoma hepaticum	Schweren Leberfunktionsstörungen mit Bewusstseinseintrübung (Coma oder Praecoma hepaticum)
