# Supplementary material for: Inconsistencies and Ambiguities in Liver-Disease-Related Contraindications—A Systematic Analysis of SmPCs/PI of Major Drug Markets
Source: J Clin Med. 2022 Mar 30;11(7):1933. doi: 10.3390/jcm11071933 (PMC9000103; doi:10.3390/jcm11071933)
Supplement: Supplementary file 1 [file jcm-11-01933-s001.zip › Table S4.pdf]

Table S4: Examples of assigning conditions to different severity grades of hepatic impairment

Term	Disease/lab parameter
Severe hepatic impairment	Liver cirrhosis, Child-Pugh-Score C
Severe hepatic impairment	Acute hepatitis
Severe hepatic impairment	Serum Albumin < 25 g/l
Severe hepatic impairment	Coma/Precoma hepaticum
Moderate hepatic impairment	Liver cirrhosis, Child-Pugh-Score B
Mild hepatic impairment	Liver cirrhosis, Child-Pugh-Score A

Further, often used combinations of terms in SmPCs/PI might be added to the list, when a consensus for severity is established. For example, 'active liver disease or unexplained persistent elevations of serum transaminases exceeding 3 times the upper limit of normal' could be used as a lab value (serum transaminases, > 3 x ULN) for defining 'active liver disease'. Still there is uncertainty to what conditions 'unexplained and persistent' includes.
