# Supplementary material for: Inconsistencies and Ambiguities in Liver-Disease-Related Contraindications—A Systematic Analysis of SmPCs/PI of Major Drug Markets
Source: J Clin Med. 2022 Mar 30;11(7):1933. doi: 10.3390/jcm11071933 (PMC9000103; doi:10.3390/jcm11071933)
Supplement: Supplementary file 1 [file jcm-11-01933-s001.zip › Document S1.pdf]

Document S1: Exclusion criteria of drug selection

UKJ all pharmaceuticals 2019, exclusion criteria:

- stomatological preparations (ATC code A01)
- digestives incl. enzymes (ATC code A09)
- insulines (ATC code A10A)
- vitamins (ATC code A11)
- mineral supplements (ATC code A12)
- other alimentary tract and metabolism products (ATC code A16A)
- local hemostatics (ATC code B02BC)
- i.v. solution additives (ATC code B05X)
- agents for treatment of hemorrhoids and anal fissures for topical use (ATC code C05A)
- heparins or heparinoids for topical use (ATC code C05BA)
- dermatologicals (ATC code D)
- topical products for joint and muscular pain (ATC code M02)
- anesthetics, local (ATC code N01B)
- decongestants and other nasal preparations for topical use (ATC code R01A)
- throat preparations (ATC code R02)
- drugs for obstructive airway diseases, inhalants (ATC code R03A and R03B)
- ophtalmologicals (ATC code S01), otologicals (ATC code S02); ophtalmological and otological preparations (ATC code S03)
- immunoglobulins, normal human (ATC code J06BA)
- vaccines (ATC code J07)
- Varia:
  - V01 allergens
  - V03AC iron chelating agents
  - V03AK tissue adhesives
  - V03AM drugs for embolisation
  - V03AN medical gases
  - V03AZ nerve depressants
  - V04 diagnostic agents (except releasing hormones, gonadorelin)
  - V06 general nutrients
  - V07 all other non-therapeutic products
  - V08 contrast media
  - V09 diagnostic radiopharmaceuticals
  - V10 therapeutic radiopharmaceuticals
  - V20 surgical dressings
  - V60 homeopathics
  - V70 formulas
- veterinary drugs
- topical drugs
- herbal drugs
- antidotes

German Drug Prescription Report/Arzneiverordnungsreport (AVR) 2020:

Included categories:

- ACE-inhibitors (monopreparation)
- angiotensin receptor agonists (monopreparation)
- opioid analgesics
- non-opioid analgesics
- H1-antihistamines
- Anti-dementia drugs
- sulfonylureas
- other antidiabetics
- antiemetics and antinauseants
- traditional antiepileptics
- new antiepileptics
- alpha-adrenoreceptor blocking agents and vasodilators
- antithrombotic agents and antihemorrhagics
- nonsteroidal antiinflammatory drugs
- beta-adrenoreceptor blocking agents
- bronchospasmolytic and antiasthmatic agents, except inhalants
- calcium channel blockers
- corticosteroids
- thiazides and thiazid analogs
- high-ceiling diuretics
- aldosterone antagonists
- antigout preparations
- cardiac therapeutics
- hypnotics and sedatives
- statins
- fibrates
- proton pump inhibitors
- spasmolytics, prokinetics and carminatives
- inflammatory bowel disease therapeutics
- anti-parkinson drugs
- psychotropic drugs
- thyroid therapy
- urologicals
- oncologics

Excluded Categories:

- ACE-inhibitors (combipreparation) – most often used pharmaceuticals already in list
- angiotensin receptor agonists (combipreparation) – most often used pharmaceuticals already in list
- renin-inhibitors – DDD <20Mio.
- allergens
- antianemic preparations
- antibiotics and antiinfektives – DDD <20Mio.
- beta-adrenoreceptor blocking agents (combipreparation)
- antisympathotonics - DDD <20Mio.
- antitussives and expectorants

- dermatologicals
- thiazides (combipreparations)
- pituitary- and hypothalamic hormones
- immunglobulines and immunosuppressives
- liver therapeutics
- pancreatic enzymes
- laxatives
- antimigraine preparations – DDD <20Mio.
- immunotherapy for multiple sclerosis – DDD <20Mio.
- muscle relaxants for multiple sclerosis therapy - <20Mio DDD or topic application
- ophtalmologicals
- osteoporosis therapyr
- nasal and otological preparations
- sex hormones
- vitamines and mineral supplements
- dental drug preparations

UK prescription cost analysis 2019, exclusion criteria:

- oral solutions
- creams/topic application/inhaler
- enteral nutrition
- emollients
- other emollient preparations
- lactulose
- other food for special diet preparations
- other food preparations
- other base/diluent/suspending agent/stabiliser preparations
- other appliances
- emollient bath and shower preparations
- gluten free bread
- glucose blood testing reagents
- other toiletry preparations
- coal tar
- other purified water preparations
- light liquid paraffin
- skin Fillers And Protectives
- gluten free mixes
- urea
- zinc oxide
- alginic acid compound preparations
- ferrous fumarate
- folic acid
- ferrous sulfate
- macrogol 3350
- thiamine hydrochloride
- pancreatin
- vitamin B compound
- dimeticone (barrier)
- sodium fluoride
- colecalciferol
- senna
- chlorhexidine gluconate
- benzydamine hydrochloride
- swabs
- ispaghula husk

US TOP300 prescription drugs 2018, exclusion criteria:

- insulines
- sex hormones (hormonal contraceptives, oral use/hormones)
- vitamines, mineral supplements, electrolytes
- inhalants/topic use preparations
- polyethylene glycol 3350
- sucralfate
- peptides
- sennosides
- mouthrinse
- pancreatic lipase
