# Supplementary material for: Inconsistencies and Ambiguities in Liver-Disease-Related Contraindications—A Systematic Analysis of SmPCs/PI of Major Drug Markets
Source: J Clin Med. 2022 Mar 30;11(7):1933. doi: 10.3390/jcm11071933 (PMC9000103; doi:10.3390/jcm11071933)
Supplement: Supplementary file 1 [file jcm-11-01933-s001.zip › Table S1.pdf]

Table S1: Selection of drugs for analysis

Final list of active ingredients chosen for the analysis, drug markets where products were available and SmPC/PI source

Drug	Drug Market	SmPC/PI Source
Acetylsalicylic acid	CH	compendium.ch
Acetylsalicylic acid	DE	pharmnet.bund
Acetylsalicylic acid	UK	electronic medicines compendium (emc)
Acetylsalicylic acid	US	www.drugs.com/pro/aspirin.html
Agomelatine	CH	compendium.ch
Agomelatine	DE	fachinfo.de
Agomelatine	UK	electronic medicines compendium (emc)
Alfuzosin	CH	compendium.ch
Alfuzosin	DE	pharmnet.bund
Alfuzosin	UK	electronic medicines compendium (emc)
Alfuzosin	US	www.drugs.com/pro/alfuzosin.html
Amoxicillin/clavulanic acid	CH	compendium.ch
Amoxicillin/clavulanic acid	DE	pharmnet.bund
Amoxicillin/clavulanic acid	UK	electronic medicines compendium (emc)
Amoxicillin/clavulanic acid	US	www.drugs.com/pro/amoxicillin-and-clavulanate-tablets.html
Apixaban	CH	compendium.ch
Apixaban	DE	fachinfo.de
Apixaban	UK	electronic medicines compendium (emc)
Apixaban	US	www.drugs.com/pro/apixaban.html
Atorvastatin	CH	compendium.ch
Atorvastatin	DE	pharmnet.bund
Atorvastatin	UK	electronic medicines compendium (emc)
Atorvastatin	US	www.drugs.com/pro/atorvastatin.html
Budesonide	CH	compendium.ch
Budesonide	DE	pharmnet.bund
Budesonide	UK	electronic medicines compendium (emc)
Budesonide	US	www.drugs.com/pro/budesonide-er-tablets.html

Bupropion	CH	compendium.ch
Bupropion	DE	pharmnet.bund
Bupropion	UK	electronic medicines compendium (emc)
Bupropion	US	www.drugs.com/pro/bupropion.html
Candesartan	CH	compendium.ch
Candesartan	DE	pharmnet.bund
Candesartan	UK	electronic medicines compendium (emc)
Candesartan	US	www.drugs.com/pro/candesartan.html
Carbamazepine	CH	compendium.ch
Carbamazepine	DE	pharmnet.bund
Carbamazepine	UK	electronic medicines compendium (emc)
Carbamazepine	US	www.drugs.com/pro/carbamazepine.html
Carbidopa/Levodopa/Entacapone	CH	compendium.ch
Carbidopa/Levodopa/Entacapone	DE	fachinfo.de
Carbidopa/Levodopa/Entacapone	UK	electronic medicines compendium (emc)
Carbidopa/Levodopa/Entacapone	US	www.drugs.com/pro/carbidopa-levodopa-and-entacapone.html
Carvedilol	CH	compendium.ch
Carvedilol	DE	pharmnet.bund
Carvedilol	UK	electronic medicines compendium (emc)
Carvedilol	US	www.drugs.com/pro/carvedilol.html
Celecoxib	CH	compendium.ch
Celecoxib	DE	pharmnet.bund
Celecoxib	UK	electronic medicines compendium (emc)
Celecoxib	US	www.drugs.com/pro/celecoxib.html
Certoparin sodium	DE	pharmnet.bund
Chlortalidone	DE	pharmnet.bund
Chlortalidone	UK	electronic medicines compendium (emc)
Chlortalidone	US	www.drugs.com/pro/chlorthalidone.html
Clarithromycin	CH	compendium.ch
Clarithromycin	DE	pharmnet.bund
Clarithromycin	UK	electronic medicines compendium (emc)
Clarithromycin	US	www.drugs.com/pro/clarithromycin.html
Clonazepam	CH	compendium.ch
Clonazepam	DE	pharmnet.bund

Clonazepam	UK	electronic medicines compendium (emc)
Clonazepam	US	www.drugs.com/pro/clonazepam.html
Clopidogrel	CH	compendium.ch
Clopidogrel	DE	pharmnet.bund
Clopidogrel	UK	electronic medicines compendium (emc)
Clopidogrel	US	www.drugs.com/pro/clopidogrel.html
Clozapine	CH	compendium.ch
Clozapine	DE	pharmnet.bund
Clozapine	UK	electronic medicines compendium (emc)
Clozapine	US	www.drugs.com/pro/clozapine.html
Levodopa/Benserazide	CH	compendium.ch
Levodopa/Benserazide	DE	pharmnet.bund
Levodopa/Benserazide	UK	electronic medicines compendium (emc)
Cotrimoxazole (Trimethoprim/Sulfamethoxazole)	CH	compendium.ch
Cotrimoxazole (Trimethoprim/Sulfamethoxazole)	DE	pharmnet.bund
Cotrimoxazole (Trimethoprim/Sulfamethoxazole)	UK	electronic medicines compendium (emc)
Cotrimoxazole (Trimethoprim/Sulfamethoxazole)	US	www.drugs.com/pro/sulfamethoxazole-and-trimethoprim.html
Dabigatran etexilate	CH	compendium.ch
Dabigatran etexilate	DE	fachinfo.de
Dabigatran etexilate	UK	electronic medicines compendium (emc)
Dabigatran etexilate	US	www.drugs.com/pro/dabigatran-capsules.html
Diazepam	CH	compendium.ch
Diazepam	DE	pharmnet.bund
Diazepam	UK	electronic medicines compendium (emc)
Diazepam	US	www.drugs.com/pro/diazepam.html
Diclofenac	CH	compendium.ch
Diclofenac	DE	pharmnet.bund
Diclofenac	UK	electronic medicines compendium (emc)
Diclofenac	US	www.drugs.com/pro/diclofenac.html
Dimenhydrinate	DE	pharmnet.bund
Dimenhydrinate	US	www.drugs.com/pro/dimenhydrinate.html
Domperidone	CH	compendium.ch

Domperidone	DE	pharmnet.bund
Domperidone	UK	electronic medicines compendium (emc)
Doxycycline	CH	compendium.ch
Doxycycline	DE	pharmnet.bund
Doxycycline	UK	electronic medicines compendium (emc)
Doxycycline	US	www.drugs.com/pro/doxycycline.html
Duloxetine	CH	compendium.ch
Duloxetine	DE	fachinfo.de
Duloxetine	UK	electronic medicines compendium (emc)
Duloxetine	US	www.drugs.com/pro/duloxetine-capsules.html
Edoxaban	CH	compendium.ch
Edoxaban	DE	manufacturers homepage
Edoxaban	UK	electronic medicines compendium (emc)
Edoxaban	US	www.drugs.com/pro/savaysa.html
Eplerenone	CH	compendium.ch
Eplerenone	DE	pharmnet.bund
Eplerenone	UK	electronic medicines compendium (emc)
Eplerenone	US	www.drugs.com/pro/eplerenone.html
Etoricoxib	CH	compendium.ch
Etoricoxib	DE	pharmnet.bund
Etoricoxib	UK	electronic medicines compendium (emc)
Ezetimibe	CH	compendium.ch
Ezetimibe	DE	pharmnet.bund
Ezetimibe	UK	electronic medicines compendium (emc)
Ezetimibe	US	www.drugs.com/pro/ezetimibe.html
Fenofibrate	CH	compendium.ch
Fenofibrate	DE	pharmnet.bund
Fenofibrate	UK	electronic medicines compendium (emc)
Fenofibrate	US	www.drugs.com/pro/fenofibrate-tablets.html
Furosemide	CH	compendium.ch
Furosemide	DE	pharmnet.bund
Furosemide	UK	electronic medicines compendium (emc)
Furosemide	US	www.drugs.com/pro/furosemide.html
Glimepiride	CH	compendium.ch

Glimepiride	DE	pharmnet.bund
Glimepiride	UK	electronic medicines compendium (emc)
Glimepiride	US	www.drugs.com/pro/glimepiride.html
Hydrochlorothiazide	CH	compendium.ch
Hydrochlorothiazide	DE	pharmnet.bund
Hydrochlorothiazide	US	www.drugs.com/pro/hydrochlorothiazide.html
Ibuprofen	CH	compendium.ch
Ibuprofen	DE	pharmnet.bund
Ibuprofen	UK	electronic medicines compendium (emc)
Ibuprofen	US	www.drugs.com/pro/ibuprofen.html
Indapamide	CH	compendium.ch
Indapamide	DE	pharmnet.bund
Indapamide	UK	electronic medicines compendium (emc)
Indapamide	US	www.drugs.com/pro/indapamide.html
Ivabradine	CH	compendium.ch
Ivabradine	DE	pharmnet.bund
Ivabradine	UK	electronic medicines compendium (emc)
Ivabradine	US	www.drugs.com/pro/corlanor.html
Lercanidipine	CH	compendium.ch
Lercanidipine	DE	pharmnet.bund
Lercanidipine	UK	electronic medicines compendium (emc)
Losartan	CH	compendium.ch
Losartan	DE	pharmnet.bund
Losartan	UK	electronic medicines compendium (emc)
Losartan	US	www.drugs.com/pro/losartan.html
Melperone	DE	pharmnet.bund
Mesalazine	CH	compendium.ch
Mesalazine	DE	pharmnet.bund
Mesalazine	UK	electronic medicines compendium (emc)
Mesalazine	US	www.drugs.com/pro/mesalamine-delayed-release-tablets.html
Metamizole	CH	compendium.ch
Metamizole	DE	pharmnet.bund
Metformin	CH	compendium.ch
Metformin	DE	pharmnet.bund

Metformin	UK	electronic medicines compendium (emc)
Metformin	US	www.drugs.com/pro/metformin.html
Methotrexate	CH	compendium.ch
Methotrexate	DE	pharmnet.bund
Methotrexate	UK	electronic medicines compendium (emc)
Methotrexate	US	www.drugs.com/pro/methotrexate-sodium.html
Midazolam	CH	compendium.ch
Midazolam	DE	pharmnet.bund
Morphine	CH	compendium.ch
Morphine	DE	pharmnet.bund
Morphine	UK	electronic medicines compendium (emc)
Morphine	US	www.drugs.com/pro/morphine-extended-release-capsules.html
Naproxen	CH	compendium.ch
Naproxen	DE	pharmnet.bund
Naproxen	UK	electronic medicines compendium (emc)
Naproxen	US	www.drugs.com/pro/naproxen-tablets.html
Nebivolol	CH	compendium.ch
Nebivolol	DE	pharmnet.bund
Nebivolol	UK	electronic medicines compendium (emc)
Nebivolol	US	www.drugs.com/pro/nebivolol.html
Nimodipine	CH	compendium.ch
Nimodipine	DE	pharmnet.bund
Nimodipine	UK	electronic medicines compendium (emc)
Nimodipine	US	www.drugs.com/pro/nimodipine.html
Nitrazepam	CH	compendium.ch
Nitrazepam	DE	pharmnet.bund
Nitrazepam	UK	electronic medicines compendium (emc)
Nitrofurantoin	CH	compendium.ch
Nitrofurantoin	DE	pharmnet.bund
Nitrofurantoin	UK	electronic medicines compendium (emc)
Nitrofurantoin	US	www.drugs.com/pro/nitrofurantoin-capsules.html
Oxycodone/Naloxone	CH	compendium.ch
Oxycodone/Naloxone	DE	pharmnet.bund

Oxycodone/Naloxone	UK	electronic medicines compendium (emc)
Phenprocoumon	CH	compendium.ch
Phenprocoumon	DE	pharmnet.bund
Pravastatin	CH	compendium.ch
Pravastatin	DE	pharmnet.bund
Pravastatin	UK	electronic medicines compendium (emc)
Pravastatin	US	www.drugs.com/pro/pravastatin.html
Progesterone	CH	compendium.ch
Progesterone	DE	pharmnet.bund
Progesterone	UK	electronic medicines compendium (emc)
Progesterone	US	www.drugs.com/pro/progesterone.html
Propiverine	DE	pharmnet.bund
Propiverine	UK	electronic medicines compendium (emc)
Ranolazine	CH	compendium.ch
Ranolazine	DE	pharmnet.bund
Ranolazine	UK	electronic medicines compendium (emc)
Ranolazine	US	www.drugs.com/pro/ranolazine-er-tablets.html
Rifampicin	CH	compendium.ch
Rifampicin	DE	pharmnet.bund
Rifampicin	UK	electronic medicines compendium (emc)
Rifampicin	US	www.drugs.com/pro/rifampin-capsules.html
Rivaroxaban	CH	compendium.ch
Rivaroxaban	DE	pharmnet.bund
Rivaroxaban	UK	electronic medicines compendium (emc)
Rivaroxaban	US	www.drugs.com/pro/xarelto.html
Rosuvastatin	CH	compendium.ch
Rosuvastatin	DE	pharmnet.bund
Rosuvastatin	UK	electronic medicines compendium (emc)
Rosuvastatin	US	www.drugs.com/pro/rosuvastatin-tablets.html
Sacubitril/Valsartan	CH	compendium.ch
Sacubitril/Valsartan	DE	fachinfo.de
Sacubitril/Valsartan	UK	electronic medicines compendium (emc)
Sacubitril/Valsartan	US	www.drugs.com/pro/entresto.html
Sildenafil	CH	compendium.ch

Sildenafil	DE	fachinfo.de
Sildenafil	UK	electronic medicines compendium (emc)
Sildenafil	US	www.drugs.com/pro/sildenafil-tablets.html
Simvastatin	CH	compendium.ch
Simvastatin	DE	pharmnet.bund
Simvastatin	UK	electronic medicines compendium (emc)
Simvastatin	US	www.drugs.com/pro/simvastatin.html
Sitagliptin/Metformin	CH	compendium.ch
Sitagliptin/Metformin	DE	pharmnet.bund
Sitagliptin/Metformin	UK	electronic medicines compendium (emc)
Sitagliptin/Metformin	US	www.drugs.com/pro/janumet.html
Solifenacin	CH	compendium.ch
Solifenacin	DE	pharmnet.bund
Solifenacin	UK	electronic medicines compendium (emc)
Solifenacin	US	www.drugs.com/pro/solifenacin-tablets.html
Tamsulosin	CH	compendium.ch
Tamsulosin	DE	pharmnet.bund
Tamsulosin	UK	electronic medicines compendium (emc)
Tamsulosin	US	www.drugs.com/pro/tamsulosin.html
Ticagrelor	CH	compendium.ch
Ticagrelor	DE	fachinfo.de
Ticagrelor	UK	electronic medicines compendium (emc)
Ticagrelor	US	www.drugs.com/pro/ticagrelor.html
Tilidine/Naloxone	DE	pharmnet.bund
Torasemide	CH	compendium.ch
Torasemide	DE	pharmnet.bund
Torasemide	UK	electronic medicines compendium (emc)
Torasemide	US	www.drugs.com/pro/torsemide.html
Valproic acid	CH	compendium.ch
Valproic acid	DE	pharmnet.bund
Valproic acid	UK	electronic medicines compendium (emc)
Valproic acid	US	www.drugs.com/pro/valproate.html
Valsartan	CH	compendium.ch
Valsartan	DE	pharmnet.bund

Valsartan	UK	electronic medicines compendium (emc)
Valsartan	US	www.drugs.com/pro/valsartan-tablets.html
Valsartan/Amlodipine	CH	compendium.ch
Valsartan/Amlodipine	DE	pharmnet.bund
Valsartan/Amlodipine	UK	electronic medicines compendium (emc)
Valsartan/Amlodipine	US	www.drugs.com/pro/amlodipine-and-valsartan.html
Warfarin	DE	pharmnet.bund
Warfarin	UK	electronic medicines compendium (emc)
Warfarin	US	www.drugs.com/pro/warfarin.html
Xipamide	DE	pharmnet.bund
Xipamide	UK	electronic medicines compendium (emc)
Zolpidem	CH	compendium.ch
Zolpidem	DE	pharmnet.bund
Zolpidem	UK	electronic medicines compendium (emc)
Zolpidem	US	www.drugs.com/pro/zolpidem.html
Zopiclone	CH	compendium.ch
Zopiclone	DE	pharmnet.bund
Zopiclone	UK	electronic medicines compendium (emc)
