# Supplementary material for: Inconsistencies and Ambiguities in Liver-Disease-Related Contraindications—A Systematic Analysis of SmPCs/PI of Major Drug Markets
Source: J Clin Med. 2022 Mar 30;11(7):1933. doi: 10.3390/jcm11071933 (PMC9000103; doi:10.3390/jcm11071933)
Supplement: Supplementary file 1 [file jcm-11-01933-s001.zip › Table S2.pdf]

Table S2: Category names and terms that were assigned to the respective categories. Complete list (English and German)

Category	Terms
hepatic impairment	Leberfunktionsstörung, Funktionsstörung der Leber, hepatische Funktionsstörung, Beeinträchtigung der Leberfunktion, Eingeschränkte/Einschränkung der Leberfunktion, Leberfunktionseinschränkung, Leberfunktionsabnormität, Hepatopathie, hepatische Dysfunktion, hepatic dysfunction, liver dysfunction, impairment of liver function, liver function abnormality, hepatic function disorder, decompensated hepatic function, impaired hepatic function, impairment of hepatic function
liver disease	Lebererkrankung, Leberparenchymerkrankung, Lebertumore, Leberkrankheit, Leberleiden, Leberparenchymschädigung, Leberparenchymschaden, hepatic disease, hepatic damage
liver insufficiency	Leberinsuffizienz, hepatische Insuffizienz, hepatic insufficiency
liver cirrhosis	Leberzirrhose, biliäre Zirrhose, Zirrhose, cirrhosis, biliary cirrhosis, hepatic cirrhosis
transaminases	Leberenzymwerte, Serum-Transaminasen, Transaminasewerte, Serum-Transaminasekonzentration, serum transaminases
porphyria	Porphyrie
Child-Pugh	Child-Pugh (Klasse/class/Score)
precoma/coma	Coma hepaticum, Praecoma hepaticum, hepatische Encephalopathie, hepatic coma, hepatic encephalopathy, precomatose states (associated with liver cirrhosis)
jaundice	Gelbsucht, Verschlussikterus, Ikterus
hepatic failure	Leberversagen
hepatitis	Hepatitis
ascites	Aszites
albumin	Serumalbumin (Serum-Albumin), serum albumin
